# The Assessment of Physicochemical and Antimicrobial Properties of Hydrophilic Gels Containing Tetracycline Hydrochloride and Various Concentrations of Ethanol

**DOI:** 10.3390/pharmaceutics16060830

**Published:** 2024-06-19

**Authors:** Agnieszka Kostrzębska, Adam Junka, Malwina Brożyna, Witold Musiał

**Affiliations:** 1 Department of Physical Chemistry and Biophysics, Pharmaceutical Faculty, Wroclaw Medical University, Borowska 211, 50-556 Wroclaw, Poland; agnieszka.kostrzebska@umw.edu.pl; 2 Platform for Unique Models Application P.U.M.A., Department of Pharmaceutical Microbiology and Parasitology, Wroclaw Medical University, Borowska 211, 50-556 Wroclaw, Poland

**Keywords:** tetracycline hydrochloride, ethanol, *C. acnes*, acne, hydrogel, HPLC

## Abstract

The high prevalence of acne, which affects nearly 85% of adolescents and young adults, underscores the importance of exploring new therapeutic solutions. The aim of the present study was to design a stable hydrogel formulation containing tetracycline hydrochloride (TC) in the presence of ethanol at various concentration levels. The antibiotic stability was assessed over a period of 84 days using the HPLC method. The rheological properties of the formulations and their microbiological activity were also evaluated. Hydrogels without ethanol and those containing 5% and 25% alcohol showed similar rheological properties and high stability of the antibiotic throughout the observation period. The formulation with the highest ethanol content of 50% differed significantly from the others in terms of rheological properties. Although the flow and viscosity curves were like those of the other formulations, the viscosity values were significantly lower. The stability of tetracycline in this formulation was also significantly lower, and by the 84th day of observation, the concentration of the drug had decreased to almost 45% of its initial content. The formulations containing the highest concentration of ethanol displayed the highest activity against the biofilm of the acne-causing agent, *Cutibacterium acnes*. The study demonstrated the possibility of developing stable and antimicrobial effective hydrogel formulations with tetracycline and ethanol as a substance enhancing drug penetration into the hair follicles.

## 1. Introduction

Acne is a widespread skin disorder affecting up to 85% of teenagers and young adults [1,2,3,4,5]. It manifests as, among other effects, inflammation symptoms, caused by bacterial species developing intensively inside the hair follicles [2]. The causative microbial agent of acne is primarily *Cutibacterium acnes*, but also *Staphylococcus epidermidis* and *Malassezia* fungi [3,6,7,8,9,10,11,12]. Acne’s symptoms are considered mild in more than 80% of cases and require topical treatment only. The remaining 20% are acute cases, where oral medications such as antibiotics, retinoids, or hormonal agents are used, especially when the course of the disease is associated with scarring, or it results in high psychological discomfort to the patient [13,14,15,16,17]. Due to the numerous side effects of applied drugs, it is extremely important to ensure that both topical and systemic anti-acne treatments are effective and administered for the shortest possible duration. Furthermore, the increasing resistance of bacterial pathogens to commonly used antibiotics underscores the need to search for new treatment solutions [18,19,20]. 

This study investigated whether it is possible to design stable and microbiologically active topical hydrogel formulations combining tetracycline hydrochloride (TC) with ethanol. Tetracyclines are commonly used antibiotics in oral anti-acne therapy, and in topical treatment of bacterial skin dermatoses [13,21,22,23]. In addition to their antimicrobial action in topical therapy, it is possible to take advantage of their non-antibiotic anti-inflammatory action, which can have a beneficial effect on the course of treatment [13,24].

In addition to tetracycline, ethyl alcohol in various amounts was included in the evaluated formulations. Ethanol in topical preparations may be used both as a skin antiseptic and as an absorption enhancer. Numerous studies show an effect of ethanol on dermal penetration of analgesics, including morphine, fentanyl, and hormonal drugs [25,26,27,28]. Additionally, the drug accumulation and penetration into skin layers and hair follicles may be increased in the presence of ethanol, e.g., of topical minoxidil [26,29,30,31,32]. Due to its effects on skin lipids, the ethanol content may facilitate the penetration of antibiotics to the hair follicles through the sebum layers of the skin [28].

This study evaluated four preparations containing 0.2% TC and 0%, 5%, 25%, and 50% ethanol. A wide range of concentrations allowed the assessment of the effect of both low and high concentrations of ethanol on such a sensitive substance as tetracycline. The aim of this study was to evaluate the feasibility of developing an effective and stable antimicrobial formulation containing tetracycline hydrochloride in the presence of ethanol.

## 2. Materials and Methods

### 2.1. Reagents

Tetracycline hydrochloride (TC) (Sigma Aldrich, Poznan, Poland), 4-epitetracycline hydrochloride (4-ETC) (Cayman Chemical Company, Ann Arbor, MI, USA), 95% ethanol (Stanlab, Lublin, Poland), 2-amino-2-methyl-1,3-propanediol (AMPD) (Sigma Aldrich), Carbopol 980 NF; polyacrylic acid crosslinked with allyl pentaerythritol (Lubrizol, Wickliffe, OH, USA), and demineralized, bi-distilled water were used to prepare the formulations. Formic acid (Sigma Aldrich), acetonitrile (Sigma Aldrich), and demineralized, bi-distilled water were used in the chromatographic analysis. The microbiological analyses performed used *C. acnes* ATCC 6919 strain (ATCC, Manassas, VA, USA), Reinforced Clostridial Agar (RCA) (Merck Milipore, Burlington, MA, USA), Brain Heart Infusion medium (BHI medium) (Graso Biotech, Jablowo, Poland), crystal violet dye (ChemPur, Piekary Slaskie, Poland), 95% ethanol (ChemPur, Piekary Slaskie, Poland).

### 2.2. Preparation of Hydrophilic Gels

A series of hydrophilic gels varying in ethanol content were prepared. The alcohol content ranged from 0% in formulation 1 to 50% in formulation 4. To prepare 100 g of each formulation, appropriate amounts of distilled water, Carbopol, 2-amino-2-methyl-1,3-propanediol, and ethanol were combined and stored for 24 h in the fridge at 5 °C. Subsequently, 0.2 g TC dissolved in 4.0 g water was added. All hydrogels were homogenized for 20 min in an Alpina MR500 automatic pharmacy mixer (Alpina Polska Sp. z o.o., Konin, Poland) at 60–90 rpm to avoid aerating the formulations. Throughout the entire research process, all preparations were stored at 5 °C in light-proof containers. To evaluate activity against bacterial biofilm, two series of hydrogels were prepared, differing only in antibiotic content: hydrogels A1–4 were free of tetracycline, while hydrogels B1–4 included the antibiotic. The detailed compositions of series 1–4, A1–4, and B1–4 are shown in Table 1.

### 2.3. Measurement of the pH of Preparations

The pH of the prepared formulations was determined using a CPC-505 pH meter (accuracy ± 0.002 pH, Elmetron Sp.j., Zabrze, Poland) and a special ERH-11S pH electrode designed for testing emulsions, gels, oils and other dense substances (Elmetron Sp.j., Zabrze, Poland). The measurements were carried out five times.

### 2.4. HPLC Analysis of TC Stability

#### 2.4.1. Samples Preparation

Concentrations of tetracycline hydrochloride and its related epimer, epitetracycline hydrochloride, contained in the preparations were analyzed by HPLC. The chromatographic analysis was carried out over an 84-day period. The samples were analyzed at equal intervals of seven days until the 56th day, and final measurements were taken on days 77th and 84th. The samples were prepared for the analysis as follows: 0.5 g of each preparation was dissolved in 99.5 g of distilled water, mixed on an Arex Digital Pro magnetic stirrer (Velp Scientifica, Usmate (MB), Italy) for 20 min at 900 rpm until the gel was completely dissolved. Eight samples of 1 mL each were taken from the obtained solutions and analyzed by HPLC.

#### 2.4.2. Chromatographic Analysis

The analyses of TC and 4-ETC were performed on a Thermo Scientific Dionex UltiMate 3000 (Thermo Scientific Dionex, Sunnyvale, CA, USA). The HPLC system consisted of a UV DAD-3000 detector, an LPG-3400SD pump module, a WPS-3000TSL autosampler module, and a TCC-3000SD column oven. Chromatographic separations of the samples were performed on a RP-18 LiChroCART column, 125 mm × 3 mm, 5 μm (Merck, Darmstadt, Germany) at 40 °C. HPLC analysis of both substances was carried out using the same analytical method. The composition of the mobile phase was 0.1% formic acid in water (A) and 0.1% formic acid in acetonitrile (B). The flow rate was 1.0 mL/min with the following gradient elution: beginning with 7% mobile phase B and continuing for 0.5 min, achieving 50% at 4 min and 95% at 4.5 min, and maintaining for 1 min. From 5.5 min, the gradient was reverted to 7% mobile phase B and stopped after 7 min at this concentration. The injection volume of the sample was 10 μL. The retention time for TC was 3.34 min. Detection was conducted at a 280 nm wavelength. Chromeleon v 7.2 SR5 software (Thermo Scientific Dionex, Sunnyvale, CA, USA) was used to process the raw data. By preparing a series of aqueous solutions of commercial TC, the linearity was observed between 2.89 and 16.43 μg/mL with a correlation coefficient (R) of 0.9983 (y = 0.2626x − 0.0782). The retention time for 4-ETC was 3.11 min and the detection was conducted at a 280 nm wavelength. A calibration curve was prepared in the concentration range of 2.83–15.78 μg/L, with a correlation coefficient (R) of 0.9989 (y = 0.1854x − 0.1003). This indicates a strong positive linear relationship between concentration and response, with the R^2^ value of 0.9989 suggesting that 99.89% of the variation in the response could be explained by the concentration. 

### 2.5. Assessment of the Rheological Properties of Hydrogels

Rheological properties were assessed using a rotational rheometer Brookfield DV-III+ (AMETEK Brookfield, Middleboro, MA, USA) in a cone/plate arrangement with a CP51 cone, and the data obtained were processed using Rheocalc v 3.2 for Windows software (AMETEK Brookfield, Middleboro, MA, USA). The measuring system was equipped with a thermostat to ensure a constant, controlled temperature.

Viscosity measurements were carried out at 20 °C and 37 °C. Each measurement was taken over a decade of shear rates with a pre-shear period and a stabilized reading before data collection. The shear rate increased from 0.38 s^−1^ to 3.84 s^−1^. The speed during the measurements increased from a value of 0.1 rpm to 1.0 rpm and the appropriate forward and reverse rheograms were noted. Each viscosity measurement was repeated four times.

The temperature dependence of viscosity was assessed between 20 °C and 40 °C with a constant temperature increase of 1 °C/min. Viscosity was determined at precisely defined temperature intervals with an increase of 0.5 °C at a constant shear rate of 3.84 s^−1^ and a constant rotational speed of 1.0 rpm. Each viscosity measurement was repeated three times.

### 2.6. Evaluation of Microbiological Activity of the Preparations

#### 2.6.1. *C. acnes* Biofilm Formation in a Six-Well Plate Model and Assessment of Provided Formulations’ Activity Using Cristal Violet Assay

For experimental purposes, the American Type Culture Collection (Manassas, VA, USA) collection’s strain of *C. acnes* ATCC 6919 was applied. The strain, preserved at −80 °C, was swabbed into RCA agar (Merck Milipore, Burlington, Massachusetts, USA) and cultured at 37 °C for 48 h under anaerobic conditions provided by the anaerobic GasPack (Becton Dickinson, Franklin Lakes, NJ, USA). Then, the *C. acnes* was mixed in BHI medium (Graso Biotech, Jabłowo, Poland), and the cell density was adjusted to 0.5 MacFarland equivalent turbidity using a densitometer (Densi-La-meter II, Erba LaChema, Brno, Czech Republic), and then using serial dilutions to 10^5^ cfu/mL. A quantity of 3 mL of such suspension was added to the wells of a 6-well plate (BioStar, Munchen, Germany) and incubated anaerobically for 5 days. Next, the medium was replaced with fresh one and 5 mg samples of the provided formulations were introduced to the Cell Strainer Insert (Biologix, Shawnee Mission, KS, USA). The whole setting was then incubated anaerobically for 24 h. After incubation, the wells were gently washed 2 times with sterile saline; subsequently, 2.5 mL of 0.18% Crystal Violet dye (ChemPur, Piekary Śląskie, Polska) was introduced for 20 min to the well surface. To de-attach unbound dye, 2.5 mL of 95% ethanol (ChemPur, Piekary Śląskie, Polska) was added to the well. The plate was mechanically vortexed using Schuttler MTS-4 (IKA, Königswinter, Germany) and the obtained CV-containing solution was analyzed spectrometrically using a MultiScan Go Spectrophotometer (Thermo Fischer Scientific, Waltham, MA, USA) at a wavelength of 595 nm. Each experiment was conducted with at least six repeats.

#### 2.6.2. Measurement of Viability of *C. acnes* Biofilm Cells Using a Live/Dead Biofilm Viability Kit

The procedure of biofilm culturing and exposure to formulations was performed in the same manner as the procedures described in Section 2.6.1. of Material and Methods. Following the guidelines from the manufacturer, each specimen was stained utilizing 500 μL of the Filmtracer™ Live/Dead™ Biofilm Viability Kit (Thermo Fisher Scientific, Waltham, MA, USA). The samples were incubated in darkness for 20 min, after which the staining solution was carefully removed. The samples were then washed once using water that had been sterilized through filtration. These prepared specimens were then ready for further examination. The fluorescence-based microscopic observations were conducted using an LS620 widefield fluorescent microscope (Etaluma, San Diego, CA, USA), objective ×20. At least 6 fields of vision were taken from each experimental setting. 

#### 2.6.3. Image Processing of *C. acnes* Biofilms Using ImageJ Software

The biofilm images were processed with the use of ImageJ version 8 (National Institutes of Health, Bethesda, MD, USA) software. First, red–green–blue images were split into corresponding channel sub-images and transformed into 32-bite grey types. Next, the mean grey value was obtained from these images (the following chain of commends was applied: Analyze->Set Measurements->Mean Grey Value->Measure). The obtained mean grey value can be correlated with the value of fluorescence intensity, and is defined and presented as the combined values of all pixels divided by the pixels’ number.

### 2.7. The Statistical Analysis

Statistical calculations were conducted with GraphPad Prism 8.0. (GraphPad Software, San Diego, CA, USA). The normality of distribution was checked with Shapiro–Wilk’s test, then the ANOVA test with Tukey’s multiple comparison was performed.

## 3. Results

### 3.1. pH Value of Formulations

The acrylic acid polymer was neutralized with a weak base AMPD. The ratio of base to polymer was constant in each formulation and provided a weakly acidic pH for the formulations. Increasing the ethanol content slightly raised the pH. The results are shown in Table 2.

### 3.2. Assessment of TC Stability by HPLC Analysis

**Figure 1:**
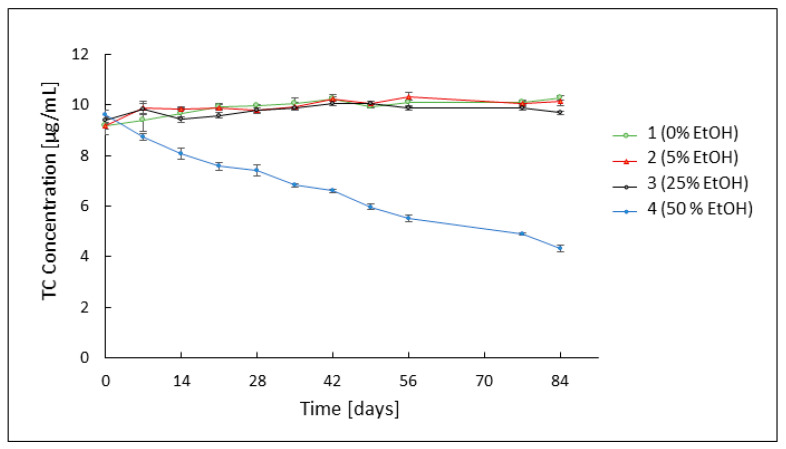
Concentration of TC over 84 days, in formulations without ethanol (1) and containing 5% (2), 25% (3), and 50% (4) of ethanol.

Chromatographic analysis of changes in the concentration of the antibiotic contained in the studied formulations over 84 days was performed to assess the effect of ethanol added to the formulations on the stability of the drug. At the same time, changes in the concentration of the tetracycline epimer, 4-epitetracycline, were observed. The analysis indicated the very high stability of tetracycline hydrochloride in formulations 1–3 throughout the observation period, i.e., almost three months. Formulations 2 containing 5% and 3 containing 25% ethanol showed drug stability comparable to that of formulation 1 without ethanol. The concentration of tetracycline hydrochloride, which on the first day of measurements was on average 9.25 μg/mL for these preparations, did not change noticeably until the end of the study. For formulation 4 containing 50% ethanol, rapid degradation of tetracycline hydrochloride was observed. The initial drug concentration in this hydrogel was about 9.62 μg/mL. By the 56th day of observation, there was an average decrease in TC content of 0.52 μg/mL per week. The last two measurements indicate a slight slowdown in the decay process, reaching a TC concentration of 4.32 μg/mL on the last day of the measurements. The changes in TC concentration are shown in Figure 1.

**Figure 2:**
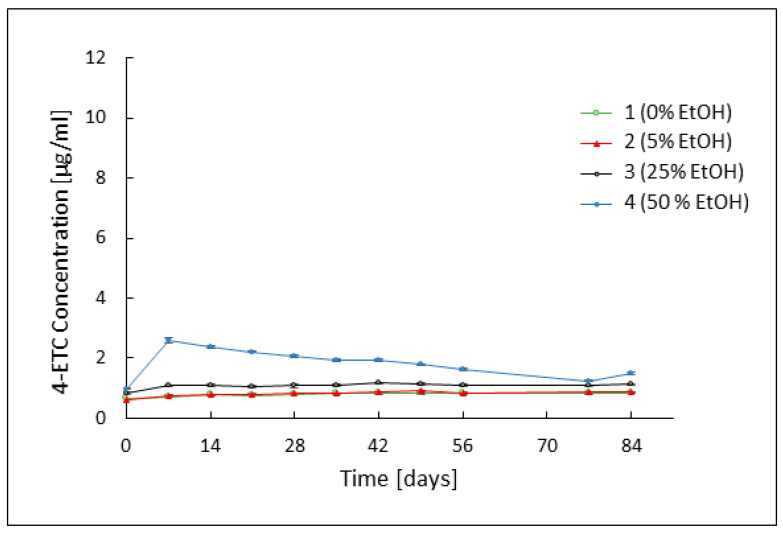
Concentration of 4-ETC over 84 days, in formulations without ethanol (1) and containing 5% (2), 25% (3), and 50% (4) of ethanol.

The same procedure applied to the tetracycline epimer concentration revealed another pattern. The 4-epitetracycline hydrochloride content in formulations 1–3 averaged 0.77 μg/mL on the first day of measurement and did not change noticeably during the study. In the case of formulation 4, an increase in the concentration of 4-ETC occurred on the 7th day of measurements, where the concentration increased from the initial value of 0.98 μg/mL to 2.59 μg/mL. A gradual decrease in epimer content was observed on subsequent days to a value of 1.51 μg/mL on the last day of measurements. Changes in 4-ETC concentration are shown in Figure 2.

### 3.3. Evaluation of the Rheological Properties of the Developed Formulations

#### 3.3.1. Viscosity Curves of Hydrogels

**Figure 3:**
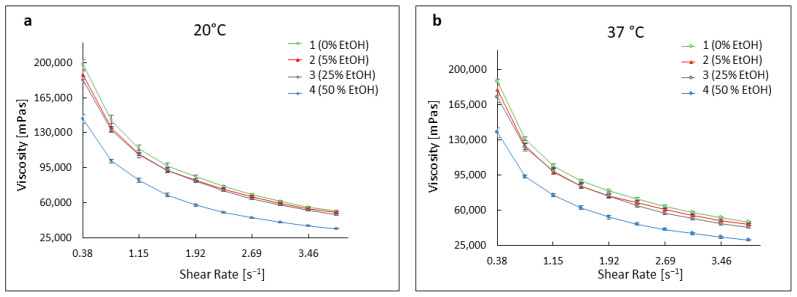
Dynamic viscosity curves for formulations in formulations without ethanol (1) and containing 5% (2), 25% (3), and 50% (4) of ethanol, at 20 °C (**a**) and 37 °C (**b**).

The developed formulations based on Carbopol serve as an example of non-Newtonian fluids that exhibit shear-thinning behavior [33,34,35,36,37]. The shear rate for all preparations was the same and increased from 0.38 s^−1^ to 3.84 s^−1^. The viscosity values for individual formulations at a temperature of 20 °C changed as follows: for gel 1, they decreased from approximately 198,000 mPas to about 51,500 mPas, for gel 2 from about 187,800 mPas to about 50,200 mPas, and for gel 3 from about 183,000 mPas to about 48,000 mPas. The viscosities of formulation 4 clearly deviated from the others, ranging from about 143,560 mPas to about 34,000 mPas. At a temperature of 37 °C, the viscosities reached slightly lower values, ranging from approximately 188,000 to 172,300 mPas for formulations 1–3, and about 137,500 mPas for formulation 4, at a shear rate of 0.38 s^−1^. These values decreased with increasing shear rate up to 3.84 s^−1^ for hydrogels 1–3, ranging from about 47,950 mPas to 43,000 mPas, and for formulation 4 to about 30,430 mPas. Figure 3 illustrates the changes in viscosity values for all formulations at a temperature of 20 °C and 37 °C.

#### 3.3.2. The Influence of Temperature on the Viscosity of the Preparations

**Figure 4:**
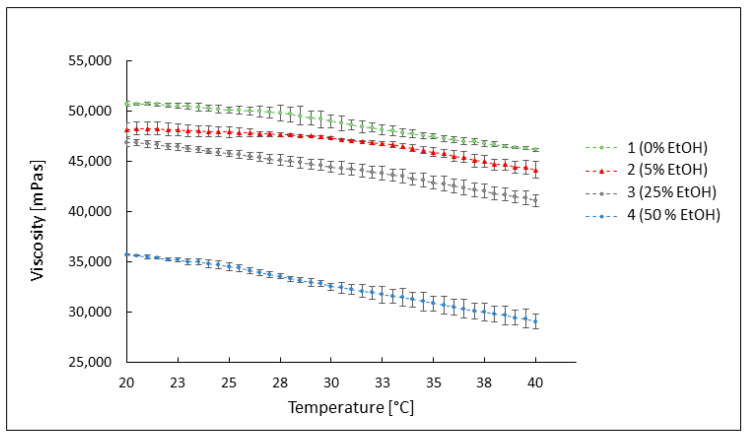
Changes in viscosity values of preparations without ethanol (1) and containing 5% (2), 25% (3), and 50% (4) of ethanol, at varying temperatures increasing from 20 °C to 40 °C.

The viscosity of all tested preparations decreased with an increase in temperature, and the reduction in viscosity in all hydrogels was slight, following a linear trend with a similar slope. Measurements were taken by increasing the temperature from 20 °C to 40 °C at a rate of 1 °C/min, with a constant shear rate of 3.84 s^−1^. For formulation 1, the viscosity decreased from approximately 51,000 mPas at 20 °C to about 46,000 mPas at 40 °C. The viscosity of formulation 2 decreased from approximately 48,100 mPas to about 44,100 mPas, and for formulation 3, viscosity decreased from ca. 47,000 mPas to ca. 41,000 mPas. Formulation 4 exhibited the lowest viscosity values, decreasing from about 35,700 to about 29,000 mPas at 40 °C. The viscosity–temperature relationship for all formulations is illustrated in Figure 4.

### 3.4. Evaluation of Microbiological Activity of the Developed Preparations

**Figure 5:**
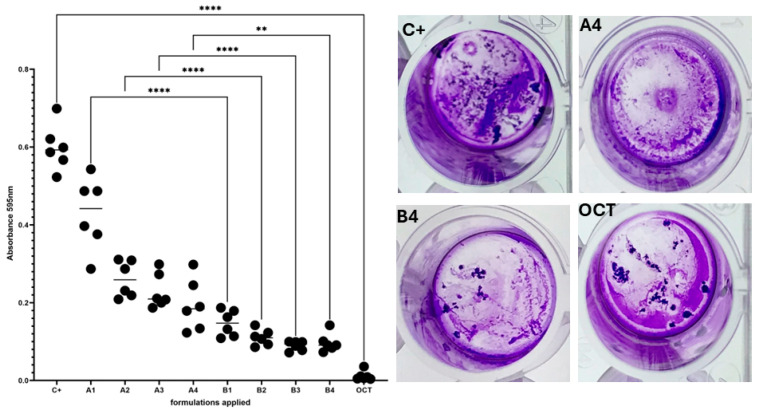
The comparison of *C. acnes* biofilm biomass exposed to the formulations A1–4, B1–4. Images: the positive control (image C+) is *C. acnes* biofilm biomass that is not exposed to the presence of any formulation, while “OCT” is the *C. acnes* biofilm biomass exposed to the topical antimicrobial agent, referred to as octenidine dihydrochloride, applied herein in the role of the experiment’s usability control (image “OCT”). Images “A4” and “B4” show CV-dyed biofilm treated with A4 or B4 formulation, respectively. The asterisks are used to show the statistical significance between the differences in biofilm biomass. (“**” indicates a significance level of 0.01, “****” indicates a significance level of 0.0001). ANOVA with Tukey’ post hoc multiple comparison test.

In the first line of evaluation of antimicrobial activity of samples provided, the standard Cristal Violet assay was performed for *C. acnes* biofilm formed on polystyrene’s surface. The results are presented in Figure 5.

**Figure 6:**
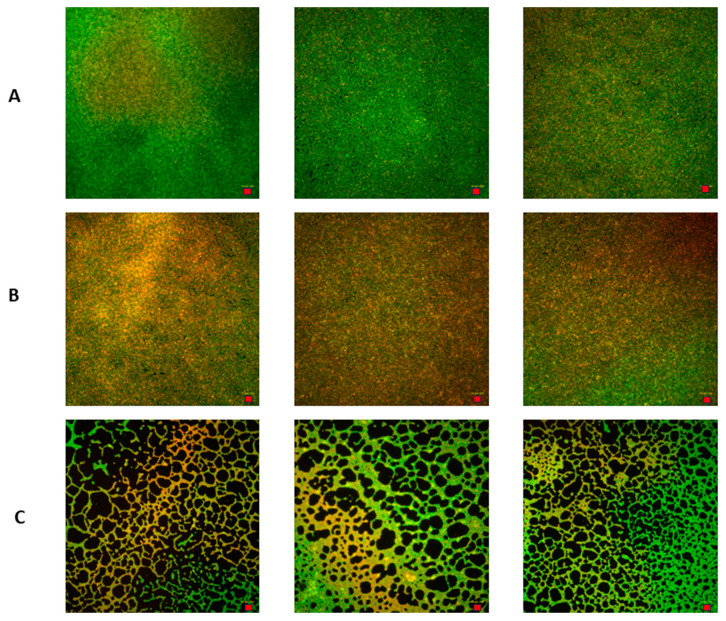
Sub-panel (**A**): images (three repeats) of non-exposed *C. acnes* biofilm (control of growth). Sub-panel (**B**): images (three repeats) of *C. acnes* exposed to formulation A4. Sub-panel (**C**): images (three repeats) of *C. acnes* exposed to formulation B4. Red bar in the lower right corner of each image represents 20 µm.

**Figure 7:**
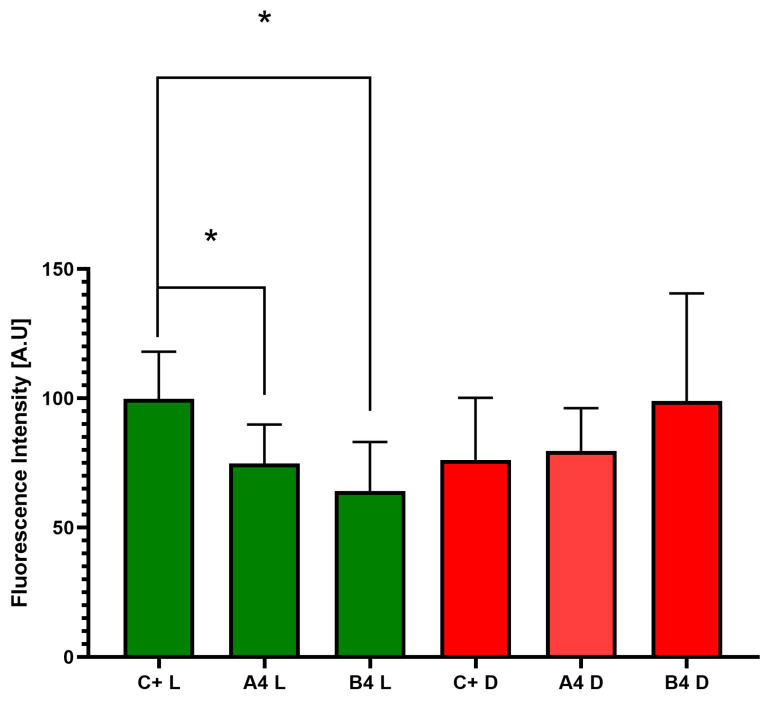
The relative number of live (L, green dots) or dead (D, red dots) biofilm-forming *C. acnes* cells exposed to A4 or B4 formulations, compared to the non-exposed *C. acnes* biofilm (C+). [A.U.]—arbitrary units. (“*” indicates a significance level of 0.01, ANOVA test).

As shown in Figure 5, all formulations from group “B” displayed a higher ability to reduce *C. acnes* biofilm biomass compared to the respective formulations from group “A”, and the difference between groups “OCT” and “C+” displayed a strong statistical difference, confirming the correctness of the experiment performed. In the next line of investigation, the *C. acnes* biofilm was exposed to the B4 or A4 formulation and dyed with the SYTO-9/propidium iodide mixture, which differentiates “live” from “dead” cells, or at least differentiates cells with intact cell walls from these with damaged cell walls. The “live” cells are dyed green, while the “dead” cells are dyed red/orange/brown. The image panel depicting differences in *C. acnes* biofilm survivability is presented in Figure 6, while quantitative data extracted from the taken images are presented in Figure 7. 

The quantitative data presented in Figure 6A reveal that the non-exposed *C. acnes* biofilm comprises a population of both live and dead cells, with a clear predominance of the former. The introduction of the A4 formulation into the experimental setup led to an increase in the population of dead cells, as indicated by the rise in red/orange coloring within the field of vision. This trend was even more pronounced with the B4 formulation, where black areas appeared in the field of vision. These areas indicate that the active ingredients in B4 eradicated the biofilm-forming cells throughout the entire structure, reaching all the way down to the abiotic polystyrene surface. The above observation was confirmed in the quantitative data shown in Figure 7. 

### 3.5. Optical Evaluation of Color Changes in Preparations

TC is a light-yellow powder and imparts a yellow color to hydrogel preparations. All formulations were characterized by a light-yellow color on the day of preparation and the first day of analysis. For formulations 1–3, the light-yellow color of the hydrogels persisted throughout the HPLC stability analysis, i.e., for 84 days. In the case of formulation 4, an intense color change process was observed, varying from red through maroon to a dark maroon, almost black color on the last day of observation. 

## 4. Discussion

Acne vulgaris is a dermatosis affecting almost 100% of the adolescent and young adult population, and treatment of acne is long-term and often requires the use of therapeutic substances with significant side effects to be successful [2,13,15]. It is therefore of crucial importance to develop pharmaceutical formulations that allow an effective and shortest possible therapy, especially with antibiotics, both topically and orally applied. 

This study was designed to evaluate the possibility of developing a topical preparation containing tetracycline hydrochloride, an antibiotic with a favorable therapeutic profile against the bacteria that cause infection in acne [21,22,23]. The aim of this study was to analyze the effect of ethanol introduction on the physicochemical and microbiological aspects of hydrophilic gels containing tetracycline hydrochloride. Hydrogel carriers, due to their physicochemical properties, are suitable for topical anti-acne therapy. They do not have a comedogenic effect and therefore should not clog hair follicles or exacerbate the course of the disease [38,39]. In the present study, ethanol was incorporated at different concentrations into hydrophilic gels containing tetracycline. Due to its properties, it can support the effective delivery of the drug deep into hair follicles colonized by inflammation-causing bacteria.

Tetracycline is a broad-spectrum antibacterial antibiotic used orally in the treatment of acne, as well as topically in dermatoses of bacterial origin [6,7,8,9]. It is known from numerous studies that tetracycline hydrochloride is a substance that is extremely sensitive to external conditions such as temperature, light exposure, or adverse pH values [40,41,42,43]. At very low pH values, below 2, tetracycline dehydrates to anhydrotetracycline (ATC), which is microbiologically inactive, while its epimer, 4-epianhydrotetracycline (4-EATC), exhibits nephrotoxic effects and can lead to reversible kidney damage known as Fanconi syndrome [41,43,44,45,46]. 4-EATC, similar to lumitetracycline, a product of light-induced tetracycline metabolism, exhibits photosensitizing effects [40,47]. In weakly acidic and nearly neutral environments, tetracycline remains very stable and slightly reversibly epimerizes to 4-ETC, which is microbiologically inactive [43,48,49]. Therefore, it was important to obtain conditions that would not lead to drug degradation. The evaluated formulations were stored at 5 °C and protected from light [50]. Previous studies have shown high stability of tetracycline hydrochloride in hydrogels with pH values not exceeding about 7.8 [51]. As tetracycline hydrochloride is most stable in a weakly acidic environment, the pH value of all formulations ranged from 6.66 to 6.95 [52]. 

Hair follicles are the important target sites for tetracycline hydrochloride. On the basis of properties of tetracycline hydrochloride, it was decided to assess whether it was possible to effectively incorporate ethanol into gel formulations containing this antibiotic as a skin permeation enhancer. Numerous studies have described the effects of ethanol on skin lipids and its properties to promote transdermal and transfollicular transport [25,26,27,28,32]. Grice et al. demonstrated in their study the favorable effects of ethanol on the penetration and accumulation of minoxidil in the hair follicles [29]. Similarly, Grams et al. noted that introducing ethanol into the formulation could improve the penetration of the drug into the hair follicle [30].

To evaluate how the amount of ethanol in the hydrogels impacts the stability of TC, all the formulations were analyzed using long-term HPLC. This analysis, which lasted almost three months, was focused on monitoring changes in the concentration of TC, as well as potential variations in the level of its derivative, 4-epitetracycline. Epimerization of tetracyclines is a natural and reversible phenomenon. The transformation product 4-epitetracycline has no antimicrobial properties and is present in low concentrations in both commercial tetracycline and in medicinal products containing the antibiotic. The epimer may revert to its parent compound or be transformed into further degradation products [42,53,54].

Stability analysis of tetracycline hydrochloride by HPLC showed very high persistence of the antibiotic in the ethanol-free hydrogel and formulations 2 and 3 containing 5% and 25% ethanol. The drug concentrations in these three formulations oscillated close to the initial value of approximately 9.25 μg/mL throughout the study. The concentration of 4-ETC also remained at the same low level throughout the observation period in all three hydrogels, indicating high stability of the system. In formulation 4 containing 50% ethanol, a rapidly progressive degradation of the antibiotic was observed during the first week of observation, accompanied by a rapid increase in the concentration of tetracycline hydrochloride epimer, despite favorable storage conditions and an appropriate pH value. On day 7 of observation, the TC concentration decreased from an initial value of almost 9.62 μg/mL to about 8.7 μg/mL. In the following weeks, a further decrease in TC concentration was observed, to a value of 4.32 μg/mL on day 84 of observation. Additionally, in the case of 4-ETC, results deviating from the other preparations were reported. On day 7 of observation, its concentration increased to a value of 2.59 μg/mL, before declining at a rate like that of the parent substance.

An additional phenomenon indicating the rapid degradation of tetracycline hydrochloride was the progressive color change of preparation 4. Tetracycline is characterized by a pale canary-yellow color, and this was the color of all preparations studied at the start of the HPLC analysis. It is known that degradation of the parent molecule can result in a degradation product having a different color to that of tetracycline. Observations of color change and darkening of tetracycline preparations have been reported in various publications, while in alkaline medium, degradation of TC to maroon quinones has been described, as pointed out by Davies and other researchers in their investigations [41,55,56]. In addition, in earlier studies, our research team noted the rapid decomposition of TC and an associated color change to dark maroon in alkaline preparations [50,51].

To incorporate ethanol into carbomer-based hydrogels, it is extremely important to select a proper carbomer neutralizing base [36]. 2-amino-2-methyl-1,3-propanediol, used in our formulations, enabled the acquisition of a stable alcohol-gel structure containing as much as 50% ethanol without the risk of carbomer precipitation. The process of gelation by neutralizing Carbopol involves the repulsion of ionic charges. During the Carbopol neutralization process, the applied AMPD causes the hydrophilic gel to swell and increase in volume as the AMPD molecule binds to the carbomer chains of the polyacrylate. The nitrogen in the 2-amino-2-methyl-1,3-propanediol molecule undergoes protonation to form the AMPD H^+^ cation, which, with weak polyacrylic acid, may form carboxylate salts [36]. The type of solvent used and its ratio to the carbomer has an influence on the rheological properties of hydrogels, their swelling, and viscosity [57,58]. Following the researchers, we speculate that the high content of polar ethanol molecules in formulation 4 may have led to ion exchange of H^+^ cations in the polymer network structure. We suspect that the high alcohol concentration of 50% may have caused ionic displacements in the microenvironment, in close proximity to the neutralized carboxyl groups of carbomer, leading to a local increase in AMPD concentration [36]. This could have resulted in accelerated decomposition of TC, which was observed during long-term HPLC analysis and manifested in the color change of the formulation to almost black on the 84th day of observation, which is characteristic for drug decomposition in an alkaline environment.

The ethanol in the hydrogels influenced both the stability of the antibiotic and the rheological and physicochemical properties of the formulations. The rheology of the studied formulations was typical for hydrogels based on acrylic acid polymer. All examined formulations exhibited characteristics of non-Newtonian viscoelastic, shear-thinning fluids [33,34,35,36,37,59,60]. The high content of the alcoholic co-solvent in formulation 4 visibly influenced the viscosity of this hydrogel. While viscosity values for formulations containing 0% to 25% ethanol were similar to each other, the viscosity of formulation 4 showed lower values. Ethanol has a higher affinity for water than Carbopol and, used at high concentrations in formulation 4, may have led to Carbopol dehydration and lower viscosity values [57,58]. Gels based on water and those with lower alcohol content exhibit greater structural rigidity and higher viscosity values. Formulation 4, containing the highest amount of ethanol, exhibited the most fluid structure of the hydrogel network. This phenomenon should be considered in the future during the potential design of pharmaceutical preparations, as excessive ethanol content can lead to the gel spreading during application, causing discomfort for the patient, as well as rapid drug release from the polymer network after application to the skin. Formulations containing a high concentration of ethanol also increased the antimicrobial activity of the TC. As the long-term stability analysis showed a progressive degradation of the antibiotic in preparation 4, to make the microbiological assessment reliable, it was carried out in the same way for all preparations, i.e., immediately after the preparation of the hydrogel. The progressive degradation of the antibiotic hinders storage of this formulation for several weeks, which encourages its development as an “ex tempore” product.

During the assessment of the temperature’s impact on the rheological properties of the hydrogel, a decrease in viscosity values for the tested formulations was observed with an increase in temperature from 20 °C to 40 °C. However, this decrease was not remarkable enough to cause discomfort during application to the skin.

The findings from this study are highly relevant to the broader context of acne treatment, particularly in the development of more effective and patient-friendly formulations. The incorporation of ethanol in hydrogels containing tetracycline hydrochloride demonstrates potential advancements in enhancing the drug’s delivery to hair follicles, a primary site of acne infection. This approach could minimize the side effects associated with systemic antibiotic treatments by enabling targeted topical therapy. Additionally, the stability and rheological properties observed in ethanol-containing formulations highlight important considerations for future pharmaceutical designs aimed at optimizing the balance between efficacy and patient comfort. The antimicrobial properties of these formulations were particularly notable, as even those with high ethanol content, which showed a progressive degradation of the antibiotic, initially exhibited enhanced antimicrobial activity against *Cutibacterium acnes* biofilm. These insights contribute to the ongoing efforts to improve acne treatment regimens, offering promising avenues for reducing treatment duration and improving patient outcomes through more stable and effective topical therapies.

## 5. Conclusions

The results suggest the potential for developing sophisticated hydrogel formulations in the future, which could integrate microbiologically effective tetracycline hydrochloride with ethanol. Ethanol, known for its impact on skin lipids, could serve as a penetration enhancer, aiding in the delivery of active ingredients deep into the skin. This could be particularly beneficial for targeting hair follicles inflamed during acne exacerbations. It appears feasible to carefully determine the concentrations of active components to preserve the stability of this sensitive antibiotic when combined with ethanol, which facilitates the antibiotic’s penetration into the hair follicles. The long-term stability of tetracycline in hydrogel formulations containing up to 25% ethanol was demonstrated. Additionally, in rheological properties, 25% ethanol concentration did not substantially affect the viscosity and swelling of the formulation. Only high ethanol content, i.e., 50%, resulted in very rapid degradation of the antibiotic and in alternations of rheological parameters. Upcoming studies are planned to microscopically assess the penetration of these formulations into the layers of human or animal skin follicles.

## Figures and Tables

**Table 1:** Composition of the formulations 1–4 and two hydrogel series prepared for microbiological analysis—formulations A1–4 were free of tetracycline, formulations B1–4 contained tetracycline.

Formulation	TC [g]	Ethanol [g]	AMPD [g]	Carbopol 980 NF [g]	Water [g]
A1	0.0	0.0	1.1	1.0	97.9
A2	0.0	5.0	1.1	1.0	92.9
A3	0.0	25.0	1.1	1.0	72.9
A4	0.0	50.0	1.1	1.0	47.9
1, B1	0.2	0.0	1.1	1.0	97.7
2, B2	0.2	5.0	1.1	1.0	92.7
3, B3	0.2	25.0	1.1	1.0	72.7
4, B4	0.2	50.0	1.1	1.0	47.7

**Table 2:** pH value of formulations 1–4, *p* < 0.0001.

Formulation	1	2	3	4
pH value	6.66 ± 0.045	6.74 ± 0.017	6.82 ± 0.007	6.95 ± 0.034

## Data Availability

Data are contained within the article.

## Abbreviations

4-EATC	4-epianhydrotetracycline
4-ETC	4-epitetracycline hydrochloride
AMPD	2-amino-2-methyl-1,3-propanediol
ATC	anhydrotetracycline
BHI	Brain Heart Infusion
C+	positive control
HPLC	High-Performance Liquid Chromatography
OCT	octenidine dihydrochloride
RCA	Reinforced Clostridial Agar
rpm	Revolutions per minute
